# Intussusception of the appendix secondary to endometriosis: a case report

**DOI:** 10.1186/1752-1947-2-12

**Published:** 2008-01-22

**Authors:** Samia Ijaz, Surjit Lidder, Waria Mohamid, Martyn Carter, Hilary Thompson

**Affiliations:** 1 Department of General Surgery, Lister Hospital, Coreys Mill Lane, Stevenage, Hertfordshire, SG1 4AB UK

## Abstract

**Introduction:**

Intussusception of the appendix is an extremely rare condition that ranges from partial invagination of the appendix to involvement of the entire colon. Endometriosis is an exceptionally rare cause of appendiceal intussusception and only very few cases have been reported in the literature to date.

**Case presentation:**

A 40 year-old woman presented to clinic with a long history of lower abdominal pain, loose motions and painful, heavy periods. Subsequent colonoscopy revealed submucosal endometriotic nodules in the sigmoid as well as a polyp thought to be arising from the appendix, which had inverted itself. She was referred to a colorectal surgeon because the polyp could not be removed endoscopically despite several attempts. At laparotomy, the appendix had intussuscepted but it was possible to reduce it and therefore a simple appendicectomy was carried out. On histology, there were widespread endometrial deposits within the wall of the appendix and this was thought to be the basis for the intussusception.

**Conclusion:**

Histological evidence of the lead point is of crucial importance in cases of appendiceal intussusception, in order to exclude an underlying neoplastic process. Consequently, surgical resection is necessary either through an open or a laparoscopic approach. Gastrointestinal endometriosis should be considered as a cause of appendiceal intussusception in post-menarchal women with episodic symptoms and proven disease.

## Introduction

Intussusception of the appendix is an extremely unusual clinical entity. A study by Collins [1] described an incidence of 0.01% based on 71,000 appendiceal specimens. The condition ranges from partial invagination of the appendix to involvement of the whole colon where the appendix may protrude from the anus [2]. It occurs predominantly in the first decade of life, with a 4:1 male to female ratio, and may be more common than traditionally believed because transient appendiceal intussusception has been reported on barium enema in asymptomatic patients [3].

The coincidence of endometriosis and intussusception is even more rare with few cases reported in the literature.

## Case presentation

**Figure 1:**
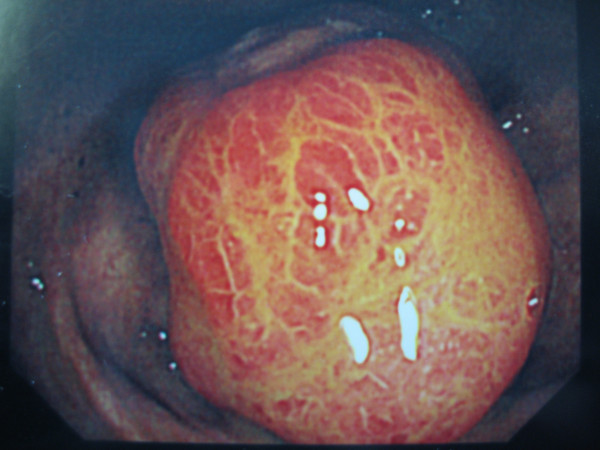
Colonoscopy view of suspected caecal polyp.

A 40-year-old woman presented to gastroenterology outpatients clinic with a several month history of right iliac fossa pain and loose motions. Apart from longstanding dysmenorrhoea and menorrhagia, she did not have any other symptoms. There was no past medical history to note and no family history of endometriosis. A clinical examination of the patient, including a full gynaecological examination, was within normal limits. Preliminary investigations revealed an iron deficiency anaemia with a haemoglobin level of 11.1 g/dl, a mean corpuscular volume of 71 fl and a low ferritin level of 8.4 ng/ml. A colonoscopy was duly organised which showed a sessile 1 cm polyp in the caecum [see figure 1]. On biopsy, this proved to be a metaplastic polyp. A subsequent attempted polypectomy was unsuccessful so the patient was referred to a tertiary centre where another attempt at polypectomy was carried out. At this point, the polyp looked to be arising from the appendix, which itself was inverted. In addition, submucosal nodules in the sigmoid were noted and these were thought to be endometrial in origin as the patient had a long history of painful and heavy periods. The polyp was not removed and the patient was referred to the colorectal surgeons and gynaecologists for a possible right hemicolectomy, total abdominal hysterectomy and bilateral salpingo-oophorectomy.

A preoperative CT scan of her abdomen and pelvis did not reveal any firm evidence of endometriosis and only noted small cysts on both ovaries.

At the time of the operation, the appendix had intussuscepted and a simple appendicectomy, rather than a right hemicolectomy, was carried out in the absence of any other findings at laparotomy.

**Figure 2:**
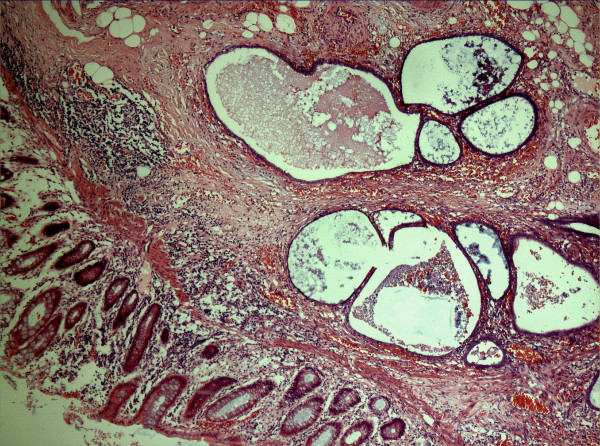
Low power (5 × 10) view of caecal wall showing endometriotic glands and stroma within the submucosa. Haematoxylin and eosin stain.

**Figure 3:**
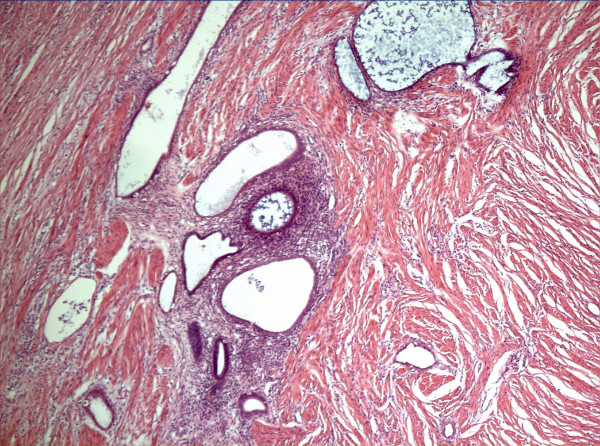
Low power (5 × 10) view of appendix wall showing foci of endometriosis within the muscle layer. Haematoxylin and eosin stain.

On histology, the wall of the appendix had widespread endometrial deposits [see Figures 2 and 3] and there was no evidence of malignancy. In addition, the cervix and fallopian tubes were within normal limits and the ovaries both had multiple follicular cysts and germinal inclusion cysts and there were leiomyomas within the myometrium.

## Discussion

Appendiceal intussusception is uncommon and typically found at the time of operation. An incidence rate of 0.01% has been reported in the literature [1]. Usually associated with males in the first decade, patients tend to present with symptoms of vague colicky lower abdominal pain with or without symptoms of small bowel obstruction.

Endometriosis is defined as the proliferation and function of endometrial tissue outside the endometrial cavity. The reported incidence in pre-menopausal women is in the order of 8–15%. Although the disease classically involves the pelvic organs and pelvic peritoneum, seeding has been observed in surgical scars, around the umbilicus, in the inguinal canal, intestines, bladder, heart and lungs. The exact aetiology of endometriosis is unknown but there are two main theories on its pathogenesis. The transportation theory presumes that endometrial cells are transported to distant sites through surgical manipulation, menstrual shedding via the fallopian tubes or through lymphatic or vascular spread. Alternatively, the metaplastic theory suggests that embryonic coelomic mesothelium dedifferentiates into endometrial tissue in response to inflammation or trauma [4,5]. The most common symptoms of endometriosis are dysmenorrhoea, pelvic pain and infertility but patients can also be asymptomatic.

The incidence of gastrointestinal endometriosis varies between 3–37% of those women who have proven disease. The rectum and sigmoid colon are most commonly involved, followed by the rectovaginal septum, small intestine, caecum and appendix. It usually takes the form of asymptomatic, small, serosal deposits. Under cyclical hormonal influences these deposits may proliferate and infiltrate the bowel wall. Cyclical haemorrhage from the endometrioma then leads to an intense, localised fibrosis within the bowel wall that can result in the formation of strictures. In addition, serosal deposits can lead to the formation of adhesions between neighbouring pelvic structures or bowel loops [6].

Appendiceal endometriosis is usually asymptomatic. When symptomatic it frequently presents as appendicitis. Acute appendiceal inflammation arises due to partial or complete luminal occlusion by the endometrioma [6]. Appendiceal intussusception secondary to endometriosis is extremely rare with fewer than 30 cases reported in the literature during the last fifty years. Endometrial involvement of the appendix is usually accompanied by chronic fibrosis, inflammation and hyperplasia or hypertrophy of the muscularis propria. This hypertrophic segment serves as a lead point for hyperperistalsis hence making it prone to intussusception particularly when combined with a fully mobile appendix that has a wide proximal lumen and a fat free mesoappendix. CT abdominal scans may demonstrate a soft tissue mass in the region of the caecum, although in this particular case the CT scan did not point towards the diagnosis.

## Conclusion

As in all cases of intussusception, the index of suspicion must be high as 90% of all intussusceptions in adults are due to an underlying neoplastic process. Intestinal endometriosis should be considered as a differential diagnosis in post-menarchal women who present with episodic gastrointestinal symptoms particularly in conjunction with gynaecological symptoms. The gold standard in the investigation of similar cases would appear to be laparoscopy or laparotomy followed by surgical resection in order to obtain histological evidence of the lead point.

## Competing interests

The author(s) declare that they have no competing interests.

## Authors' contributions

All of the named authors were involved in the preparation of this manuscript.

## Consent

Written informed consent was obtained from the patient for publication of this case report and any accompanying images. A copy of the written consent is available for review by the Editor-in-Chief of this journal.
